# Bridging Sex-Specific Differences in the CAR-Mediated Hepatocarcinogenesis of Nitrapyrin Using Molecular and Apical Endpoints

**DOI:** 10.3389/ftox.2021.766196

**Published:** 2021-10-29

**Authors:** Lynea Murphy, Matthew J. LeBaron, Kamin Johnson, Reza J. Rasoulpour, Xiujuan Wang, Jessica LaRocca

**Affiliations:** ^1^ Corteva Agriscience, Indianapolis, IN, United States; ^2^ The Dow Chemical Company, Toxicology and Environmental Research and Consulting, Midland, MI, United States

**Keywords:** CAR, mode of action, pesticide, nitrapyrin, liver tumor

## Abstract

Nitrapyrin, a nitrification inhibitor, produces liver tumors in B6C3F1 mice. In a 2-year oncogenicity study, increased incidence of mice with hepatocellular tumors was observed following exposure to 125 (females only) or 250 mg/kg/day (males and females) nitrapyrin in the diet. Previous data was generated in male mice to support a mode-of-action (MoA) characterized by constitutive androstane receptor (CAR) nuclear receptor (NR) activation, increased hepatocellular proliferation, and subsequent hepatocellular foci and tumor formation. Uncertainty as to the relevance of this MoA for females remained given the increased sensitivity to tumor formation in female mice. A targeted MoA study was conducted to evaluate CAR activation and hepatic responses in female mice treated with the female carcinogenic dose of nitrapyrin for 4 days. Nitrapyrin induced a treatment-related increase in hepatocellular hypertrophy and hepatocellular proliferation. Nitrapyrin also induced a dose-related increase in the *Cyp2b10*/CAR-associated transcript and liver weights. Nitrapyrin-induced liver weights and *Cyp2b10* gene expression for both males and females were compared to data generated from three other established CAR activators; methyl isobutyl ketone, phenobarbital, and sulfoxaflor. The response observed in female mice following exposure to nitrapyrin was within range of the degree of change observed in mice following exposure to tumorigenic doses of other CAR activators. Consistent with the liver MoA in male mice, these data support a CAR-mediated mode of action for nitrapyrin-induced liver tumors in female mice, with the understanding that a focused approach minimizing animal use can bridge male and female datasets when sex-specific carcinogenic differences are observed.

## Introduction

**FIGURE 1:**
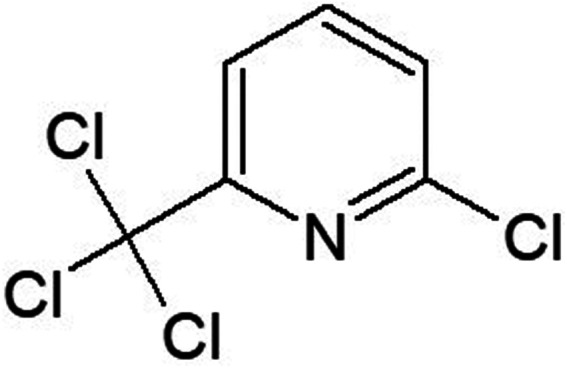
Nitrapyrin chemical structure.

Nitrapyrin (2-chloro-6-(trichloromethyl) pyridine; CAS Number 1929-82-4; Figure 1) is the active ingredient in N-SERVE^™.^ ^1^ nitrogen stabilizer. Two separate dietary chronic toxicity/2-year carcinogenicity studies were performed with a total of five dose levels of nitrapyrin up to 250 mg/kg/day in B6C3F1 mice. In the first chronic/oncogenicity study, mice were given nitrapyrin at 0, 5, 25, or 75 mg/kg/day, and in a second carcinogenicity study, male and female mice were given 0, 125 or 250 mg/kg/day nitrapyrin in the diet. At 75, 125, and 250 mg/kg/day, males and females had increased absolute and relative liver weights. Microscopic evaluation revealed hepatocellular hypertrophy and altered tinctorial properties at 125 and 250 mg/kg/day in males and females, while single cell necrosis was identified only in males at 250 mg/kg/day. An increased incidence of mice with hepatocellular tumors (adenomas and/or carcinomas) were observed following exposure to 125 (females only) or 250 mg/kg/day (males and females) nitrapyrin in the diet. Tumor incidence data have been published (LaRocca et al., 2017).

To elucidate the mechanism underlying nitrapyrin-mediated mouse liver tumors, several experiments were conducted in male mice that included apical, molecular, biochemical, and cellular endpoints. A single sex was used to minimize animal use while still comprehensively evaluating the MoA. Accordingly, the data generated in male mice were evaluated for MoA/Human Relevance Framework (HRF) and against Bradford Hill criteria and strongly support that nitrapyrin-induced mouse hepatocarcinogenesis is mediated via a CAR-NR activation MoA (Sonich-Mullin et al., 2001; Cohen et al., 2003; Meek et al., 2003; Boobis et al., 2006; Lake 2009; LaRocca et al., 2017). The MoA for CAR activation was represented by three key events. Key Event #1 (molecular initiating event) was characterized by activation of CAR leading to CYP isozyme induction. Key Event #2 was characterized as an increase in hepatocellular proliferation. Key Event #3 (adverse outcome) was characterized by subsequent induction of proliferative lesions in the liver including hepatocellular foci, adenomas, and carcinomas (LaRocca et al., 2017). However, given that only male mice were used to establish the MoA and female mice had an increase in hepatocellular adenomas at a lower dose level than males, confirmation of the CAR MoA in female mice was necessary. Therefore, a focused study was conducted to address this uncertainty and understand if the same MoA applied to females.

## Materials and Methods

### Animals and Treatment

This study used female B6C3F1 mice at one dose level of nitrapyrin (i.e., 125 mg/kg/day) and a vehicle control (*n* = 6 animals/dose). This dose level was used in acarcinogenicity study which resulted in increased incidence of liver tumors (hepatocellular adenomas) in females. Increased absolute and relative liver weights were observed in females at 125 mg/kg/day at 12 months and 2 years. Additionally, hepatocellular hypertrophy, and single cell necrosis were found in females at 2 years.

Animals were approximately 12 weeks of age at study start. This age is slightly older when compared to the starting age of mice from previously conducted studies with nitrapyrin (6–8 weeks of age at study start). The purpose for using slightly older mice was to aid in reducing the background hepatocellular proliferation that is observed with younger animals which may confound the assessment and interpretation of the hepatic proliferative response to nitrapyrin (Eldridge and Goldsworthy, 1996; Furukawa et al., 2003).

The studies were performed at The Dow Chemical Company, Toxicology and Environmental Research and Consulting (TERC), Midland, Michigan, which is accredited by the American Association for Accreditation of Laboratory Animal Care. All animal care and use activities were reviewed and approved by the Institutional Animal Care and Use Committee. Animals were housed singly in stainless steel cages with wire mesh floors and were provided non-woven gauze and a cardboard enclosure for enrichment. Animals were provided LabDiet Certified Rodent Diet #5002 (PMI Nutrition International, St. Louis, MO) *ad libitum* via a glass feed crock with a stainless steel lid. Municipal water was provided *ad libitum* by a pressure-activated lixit valve-type watering system. Water provided was periodically analyzed by both the municipality and an independent testing facility for chemical and biological contaminants which might interfere with study conduct or interpretation. Animals were housed in a room designed to provide a uniform temperature of 22°C (range of 20–26°C), uniform humidity of 50% (range of 30–70%), 10–15 air changes/hour, and a 12 h light/dark photoperiod. After acclimation for 1 week, animals were randomly assigned to exposure groups via a computer program designed to increase the probability of uniform group mean body weights and standard deviations. Animals were uniquely identified via subcutaneously implanted transponders (BioMedic Data Systems, Seaford, DE).

### Dietary Test Article Preparation and Analysis

Nitrapyrin was prepared consistent with prior studies (LaRocca, et al., 2017). Nitrapyrin was initially dissolved in acetone. The Nitrapyrin-containing diet was prepared by diluting this concentrated test material-feed mixture with ground feed. The test material was mixed as a fixed percentage in the diet. Control animals received control feed prepared using an equivalent amount of acetone representative of the amount of acetone in the premix that was used in the 125 mg/kg/day dose. After mixing with acetone, control, and nitrapyrin-containing feeds were left overnight in a vented area to volatilize the acetone before being fed to the animals. The concentration of the test material in the diet was not adjusted for purity. Dose confirmation and homogeneity analyses were not conducted; however, nitrapyrin is stable in rodent feed for at least 15-days at concentrations targeting dose levels up to 250 mg/kg/day (Stebbins and Cosse, 1997). The study was completed within 15-days of diet preparations.

### Study Duration

A treatment duration of 4 days in the diet was chosen based on optimal induction of hepatocellular proliferation and *Cyp2b10* transcript levels at 4 days from the previous nitrapyrin study in B6C3F1 and C57BL6/NTac mice (LaRocca et al., 2017). Previous studies with nitrapyrin indicated treatment-related hepatic effects in male B6C3F1 mice at 4 days with corresponding increases in liver weights, along with hepatocellular hypertrophy and hepatocellular proliferation. CAR activation was induced as demonstrated by robust increases in the *Cyp2b10*/CAR-associated transcript of 371-fold compared to controls at 4 days. In addition, a gross necropsy was conducted, and liver and kidney weights were recorded.

### Hepatocellular Proliferation

All animals were implanted with an osmotic pump containing BrdU prior to exposure. Hepatic S-phase DNA synthesis was determined using BrdU immunohistochemistry for identification of BrdU incorporation into nuclear DNA using the method outlined by Eldridge and Goldsworthy (1996). Upon euthanasia, a section of liver was recovered as noted above, processed by standard techniques, and mounted on glass slides. Tissue was stained for BrdU using the manufacturers’ protocol (BD Biosciences, San Diego, California) and modified heat-induced antigen retrieval. A small section of duodenum from each animal was also processed to serve as a control for confirming systemic availability of the BrdU as well as confirmation of BrdU immunohistochemistry by visual inspection. BrdU was not scored for the duodenum. All slides were digitized using a Versa whole-slide scanner (Leica Biosystems) and the original digital image files stored on a secure GLP compliant server (e-Slide Manager; Leica Biosystems). A labeling index (BrdU-positive hepatocytes/BrdU-positive + BrdU-negative hepatocytes x 100) was calculated for each of three hepatocellular regions: centrilobular, midzonal, and periportal using a semi-automated image analysis system (Halo; Indica Labs). At least 1,000 cells/region/animal in centrilobular, midzonal, and periportal regions were evaluated.

### Body Weights/Body Weight Gains

All animals were weighed pre-exposure (test day (TD) −4 and −1) and TD 1 and 5. Descriptive statistics (i.e., mean ± standard deviation) of body weights were performed using data collected on TD 1 and 5. Body weight gains were calculated relative to TD 1. The weight of the osmotic pump was subtracted from the body weight recorded on TD 1 and the terminal body weight to allow for accurate calculation of the body weight and body weight gains.

### Anatomic Pathology

Mice were not fasted prior to necropsy. After weighing, mice were anesthetized with a mixture of isoflurane vapors and medical oxygen and then further anesthetized with carbon dioxide. Their tracheas were exposed and clamped, and the animals were euthanized by decapitation.

After the animal was euthanized, the osmotic pump was removed and weighed individually. The weight of the osmotic pump was subtracted from the body weight recorded to get an accurate terminal body weight. The skin was then reflected from the carcass, the abdominal cavity opened, and the liver was excised. Additionally, a 2–3 cm segment of the proximal duodenum was excised, flushed with 10% buffered neutral formalin, and placed in the same fixative with the liver for analysis of BrdU incorporation. This tissue served as a positive control for BrdU uptake. The liver was trimmed, weighed, and processed as follows: the upper third of the left lateral liver lobe was processed in RNALater for targeted gene expression analysis (conducted at MPI Laboratory). Cross sections through the middle of the left lateral lobe, middle of the left and right medial lobes, and through the right lateral lobe were preserved in neutral, phosphate-buffered 10% formalin for histological evaluation and for BrdU proliferation analysis. Transponders were removed and placed in the formalin with the liver and duodenum tissue. The ratios of liver weight to terminal body weight were calculated.

The livers and duodenums from all study animals were processed by standard histologic procedures. Paraffin embedded tissues were sectioned approximately 6 µm thick, stained with hematoxylin and eosin, and examined by a board-certified veterinary pathologist using a light microscope. Selected histopathologic findings were graded to reflect the severity of specific lesions to evaluate: 1) the contribution of a specific lesion to the health status of an animal and 2) exacerbation of common naturally occurring lesions as a result of the test material. Very slight and slight grades were used for conditions that were altered from the normal textbook appearance of an organ/tissue but were of minimal severity and usually with less than 25% involvement of the tissue. The histopathologic severity grade of very slight is synonymous with minimal. This grade was used for histopathologic observations that were at the lower threshold of light microscopic recognition. The histopathologic severity grade of slight is synonymous with mild. This grade was used for histopathologic observations that were easily discernable, yet typically involved less than 25% of the tissue or specific cell type in which the observation occurred. This type of change would neither be expected to significantly affect the function of the specific organ/tissue nor have a significant effect on the overall health of the animal. Very slight or minimal centrilobular hypertrophy is typically interpreted to be non-adverse. A moderate grade was used for conditions that were of sufficient severity and/or extent (up to 50% of the tissue) that the function of the organ/tissue may have been adversely affected, but not to the point of organ failure. The health status of the animal may or may not have been affected, depending on the organ/tissue involved, but generally lesions graded as moderate would not be life threatening. A severe grade would have been used for conditions that were extensive enough to cause significant organ/tissue dysfunction or failure. This degree of change in a critical organ/tissue may have been life threatening.

### Gene Expression

Liver samples preserved in RNALater from all study animals that survived to scheduled necropsy were used for RNA isolation. Total RNA was extracted using the Qiagen RNeasy kit following the manufacturer’s protocol. RNA quantity was assessed by a NanoDrop ND-1000 spectrophotometer. Only samples with an OD 260/280 ratio greater than 1.8 and with clearly defined 28S and 18S bands were used for gene expression studies. Total RNA was treated with DNase enzyme to avoid DNA contamination. The RNA extraction samples were stored at approximately −80°C until shipment on dry ice to MPI Research, Inc. (Mattawan, MI) for gene expression analysis.

The following genes were selected to aid in understanding the potential mode of action and other possible metabolic pathways of nitrapyrin in female mice and were analyzed in a singleplex reaction. Gene expression responses for *Cyp1a1*, *Cyp2b10*, *Cyp3a11*, and *Cyp4a10* were assessed as biomarkers for activation of AhR (Whitlock, 1999), CAR (Honkakoski et al., 1998; Kawamoto et al., 1999; Wei et al., 2000), PXR (Kliewer et al., 1998), and PPAR-α (Palmer et al., 1994; Aldridge et al., 1995) signaling pathways, respectively.

1. *Cyp1a1*: “AhR response gene”. Mouse ABI TaqMan ID: Mm00487218_m1
2. *Cyp2b10*: “CAR response gene”. Mouse ABI TaqMan ID: Mm00456591_m1
3. *Cyp3a11*: “PXR response gene”. Mouse ABI TaqMan ID: Mm00731567_m1
4. *Cyp4a10*: “PPAR-α response gene”. Mouse ABI TaqMan ID: Mm01188913_gl

The following housekeeping gene was selected as endogenous reference for normalization purpose and was concurrently analyzed, in a singleplex reaction:

*Actb*: Mouse beta-Actin, endogenous control. Mouse ABI TaqMan ID: Mm00607939_s1.

### Statistics

Means and standard deviations were calculated for all continuous data. All parameters examined statistically (feed consumption is addressed below) were first tested for equality of variance using Bartlett’s test (Winer, 1971). If the results from Bartlett’s test were significant at alpha = 0.01, then the data for the parameter may be subjected to a transformation to obtain equality of the variances. The transformations that were examined were the common log, the inverse, and the square root, in that order. The data were reviewed and an appropriate form of the data was selected. The selected form of the data was then subjected to the appropriate parametric analysis as described below.

Initial body weight, terminal body weight, absolute and relative liver weights, and cell proliferation were evaluated using a *t*-test at alpha = 0.05. Descriptive statistics only (means and standard deviations) were reported for body weight gain.

Gene expression was quantified using the comparative Ct method (ΔΔCt) (Schmittgen and Livak, 2008). For this method, the amount of target mRNA for each treatment group was expressed relative to an endogenous reference gene and relative to vehicle control animals. The mRNA amounts of the selected genes were calculated using beta-actin (Actb) as endogenous reference (housekeeping gene) within each sample. The mean Ct of the housekeeping gene was subtracted from the mean Ct of the target genes (ΔCt). The mean ΔCt value from each treatment group was then subtracted from the mean ΔCt for the vehicle control group to generate the ΔΔCt value that was used to calculate the fold induction for that treatment group, relative to controls. Statistical analysis was not performed for the transcript analysis.

Because numerous measurements were statistically compared in the same group of animals, the overall false positive rate (Type I errors) was greater than the nominal alpha levels. Therefore, the final interpretation of the data considered statistical analyses along with other factors, such as the magnitude of the response and whether the results were consistent with other biological and pathological findings and historical control values.

To understand how relative liver weight change corresponded to *Cyp2b10* gene expression change, a linear model was fit for the two variables. Natural log transformation was carried out for the two variables after a nonlinear trend was observed for the original data. The analysis was done using JMP Pro version 12 software (SAS Institute Inc., Cary, NC).

## Results

### Nitrapyrin-Exposure Increased Liver Weights and Hepatocellular Hypertrophy

There were no treatment-related differences in body weights, body weight gains, or feed consumption of mice given 125 mg/kg/day compared to controls over the 4-days exposure period (data not shown). Females given 125 mg/kg/day had treatment-related higher mean absolute (14.1%) and relative (12.6%) liver weights (Table 1). All females given 125 mg/kg/day had treatment-related very slight centrilobular/midzonal hepatocellular hypertrophy with increased cytoplasmic eosinophilia (Table 2). Additionally, females had treatment-related very slight hypertrophy of the duodenal villous epithelial cells and very slight multifocal or diffuse vacuolization consistent with fatty change of the duodenal villous epithelial cellsThe duodenum was examined microscopically because it served as a positive control for BrdU immunohistochemistry. The treatment-related histopathologic effects in the liver and duodenum were consistent with previously reported nitrapyrin studies (Stebbins and Cosse, 1997; Murphy et al., 2014).

**TABLE 1:** Absolute (g) and Relative Liver Weights (g/100 g body weight) in Female B6C3F1 Mice Treated with 0 or 125 mg/kg/day Nitrapyrin for 4 days.

0 mg/kg/day (*n* = 6)		125 mg/kg/day (*n* = 6)	
Absolute Liver Wt. (g)	Relative Liver Wt. (g/100 g)	Absolute Liver Wt. (g)	Relative Liver Wt. (g/100 g)
1.258 ± 0.042	5.565 ± 0.116	**1.436** ^a^ **+/- 0.078**	**6.266** ^a^ **+/- 0.178**

Means ± standard deviations are shown.

a Statistically different from control mean by Student’s T-test, Alpha = 0.05.

Bold values were considered treatment-related effects.

**TABLE 2:** Histopathological Changes in the Liver in Female B6C3F1 Mice Treated with 0 or 125 mg/kg/day Nitrapyrin for 4 days.

Dose (mg/kg/day)	0	125
Number examinedact	6	6
Aggregates of mononuclear cells, *very slight*	1	3
Aggregates of mononuclear cells; adjacent to necrotic or degenerative hepatocytes, *very slight*	5	3
Hypertrophy; increased eosinophilia; hepatocyte; centrilobular/midzonal, *very slight*	0	**6**
Necrosis; with accompanying inflammation; hepatocyte; focal, *moderate*	0	1

Bold values were considered treatment-related effects.

One mouse had moderate focal hepatocellular necrosis with accompanying inflammation and histopathologic alterations in the gallbladder, consisting of moderate focally extensive submucosal necrosis with inflammation and slight diffuse mucosal hyperplasia. These lesions were interpreted to be spontaneous alterations, unassociated with dietary administration of nitrapyrin. The chronic nature of the gallbladder and associated liver lesions indicated that these alterations were present prior to the start of the 4-days exposure period. No other treatment-related histopathological changes were observed. The treatment-related histopathologic effects in the liver were consistent with what was previously observed in male mice exposed to nitrapyrin (LaRocca et al., 2017.)

### Nitrapyrin-Exposure Increased *Cyp2b10* Gene Expression

**FIGURE 2:**
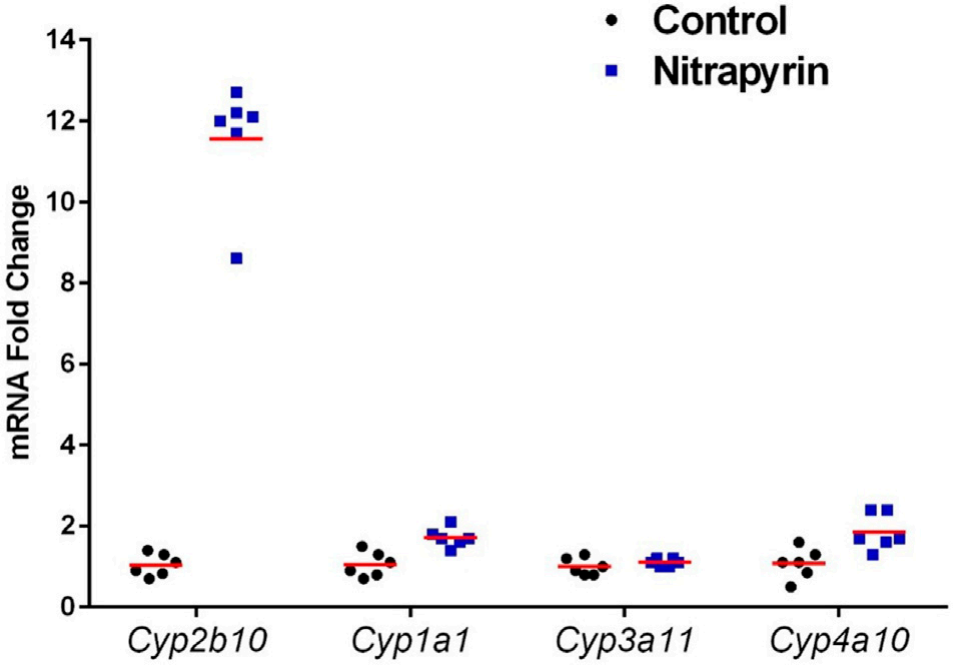
Individual Biological Replicates of Gene Expression Data in Female B6C3F1 Mice Treated with 0 or 125 mg/kg/day Nitrapyrin for 4 days. Gene expression data showing biological replicates and group means of nitrapyrin-treated female mice at 125 mg/kg/day and unexposed control mice. Each data point corresponds to the mRNA fold change for an individual animal compared to the control group mean value which was set to 1. Six animals/treatment group were examined for gene expression data.

After 4 days of 125 mg/kg/day nitrapyrin exposure, there was a treatment-related increase in *Cyp2b10* mRNA transcript levels in the liver indicating CAR activation (Figure 2). *Cyp2b10* transcript levels were 11.4-fold higher than controls. Although the magnitude of the fold-change was lower than previous nitrapyrin studies in male mice, the induction of the CAR pathway following nitrapyrin exposure was consistent. There was no biologically significant induction of *Cyp1a1*, *Cyp3a11*, or *Cyp4a10* following 4 days of exposure to nitrapyrin indicating that AhR, PXR, and PPAR-α pathways were not activated by nitrapyrin.

Nitrapyrin-exposure in mice results in a treatment-related increase in hepatocellular proliferation. Mice exposed to nitrapyrin had a slight, statistically-identified, and treatment-related increase in hepatocellular proliferation in the 125 mg/kg/day dose group as measured by an increase in BrdU labeling index (LI) in the periportal and centilobular regions along with the total labeling index compared to controls (Table 3). The LI of the midzonal region was higher than control and considered treatment-related; however, this observation was not statistically identified. Individual BrdU animal data are included in Supplementary Table S1.

**TABLE 3:** Summary of Hepatocellular Proliferation by Lobular Zones in Female B6C3F1 Mice Treated with 0 or 125 mg/kg/day Nitrapyrin for 4 days.

Dose (mg/kg/day)	CL	MZ	PP	Total
0	1	1	1	1
125	**1.34** ^a^	**1.42**	**1.45** ^a^	**1.40** ^a^

Data are fold change relative to control labeling indices.

CL = centrilobular, MZ = midzonal, PP = periportal.

Six animals/treatment group were evaluated.

Bold values were considered treatment-related effects.

a Significantly different from control mean by *t*-test, alpha = 0.05.

### Comparison of Nitrapyrin Data to Other CAR Activators in Rodents

**FIGURE 3:**
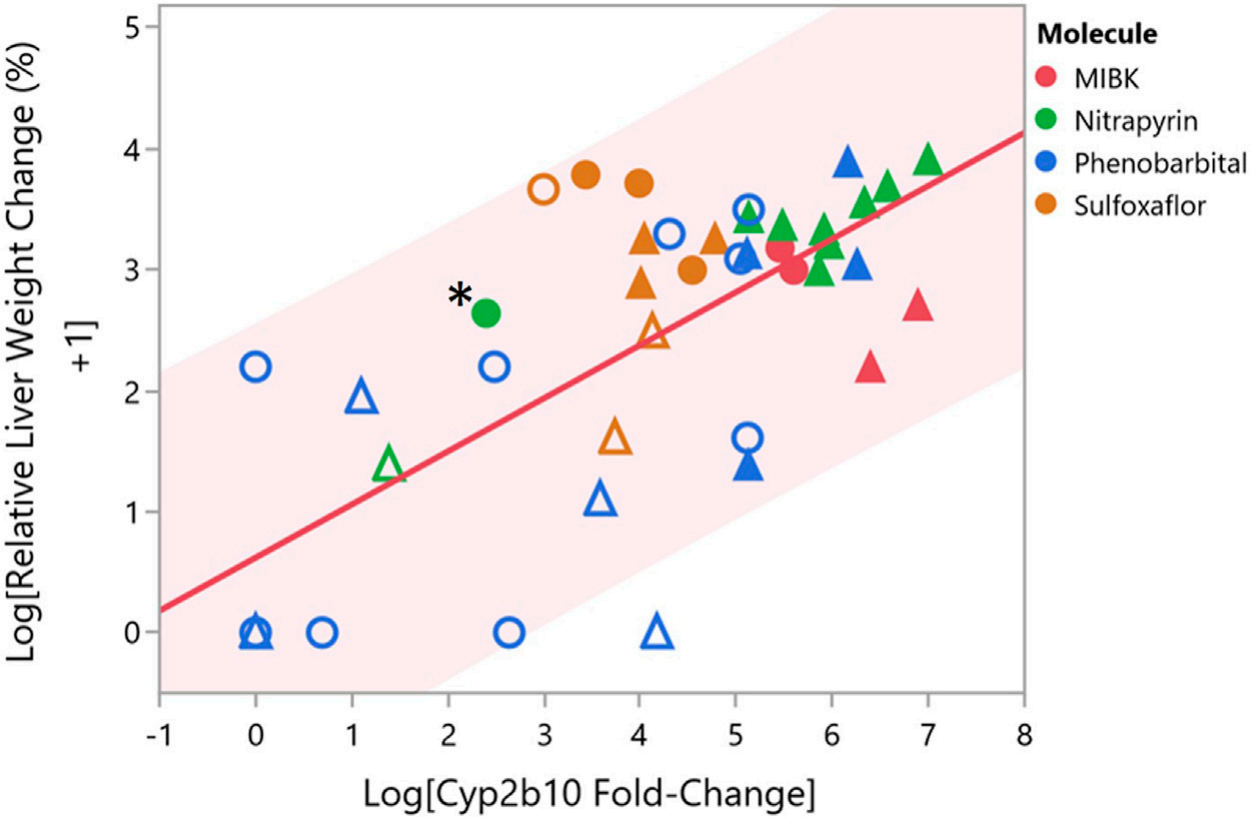
Comparison of *Cyp2b10* Gene Expression and Relative Liver Weight Changes for Male and Female Mice and Rats Following Exposure to CAR Activators Nitrapyrin, MIBK, Phenobarbital, or Sulfoxaflor. Comparison of log relative liver weight change (percent) and log *Cyp2b10* fold-change gene expression for male and female mice following exposure to either nitrapyrin (green), MIBK (red), phenobarbital (blue), or sulfoxaflor (orange) for 90-days or less. Each data point corresponds to a single dose level for one sex. Male mice are represented by triangles, and female mice are represented by circles. Data points that are filled correspond to dose levels that are at or above the tumorigenic dose for the respective molecule, and data points that are unfilled correspond to dose levels below the tumorigenic dose for the respective molecule. The data point for female mice exposed to nitrapyrin at 125 mg/kg/day is indicated by an adjacent asterisk. Shading from the trend line corresponds to the 95% prediction interval.

A wealth of dose-response data exist for *Cyp2b10* gene expression and liver weight increases, which represent necessary and associative data, respectively, of Key Event #1 for CAR activators. We therefore sought to compare the nitrapyrin data for Key Event #1 to other CAR activators using data generated within this laboratory (TERC), specifically methyl isobutyl ketone (MIBK), phenobarbital, and sulfoxaflor (Geter et al., 2010; LeBaron et al., 2013; Geter et al., 2014; Hughes et al., 2016). Phenobarbital is a standard example of a CAR activator (Whysner et al., 1996; Holsapple et al., 2006), and MIBK and sulfoxaflor have also been established as CAR activator. The dose-response relationship between relative liver weight changes and *Cyp2b10* gene expression for male and female mice and rats exposed to either MIBK, phenobarbital, sulfaxoflor, or nitrapyrin for 90-days or less are shown in Figure 3. Values shown in Figure 3 are listed in Supplementary Table S2. To understand how relative liver weight change corresponded to *Cyp2b10* gene expression change, a linear model was fit for the two variables which produced an R^2^ = 0.53, indicating the observed increases in relative liver weight and *Cyp2b10* gene expression were positively correlated. Furthermore, the *Cyp2b10* and relative liver weight data for male and female nitrapyrin-exposed mice were consistent with the other rodent CAR activators evaluated.

## Discussion and Conclusions

The MoA for CAR activation is often represented by the following key events: (Key Event #1) activation of CAR leading to CYP isozyme induction, (Key Event #2) increased hepatocellular proliferation, and (Key Event #3) subsequent induction of proliferative lesions in the liver including hepatocellular foci, adenomas, and carcinomas (adverse outcome) (Cohen, 2010; LeBaron et al., 2014; LaRocca et al., 2017). Based on previous MoA studies in male B6C3F1 mice, CAR activation was supported by a 370.7-fold increase in *Cyp2b10* gene expression and a 3.5-fold increase in panlobular hepatocellular proliferation following 4 days of exposure to the male carcinogenic dose of nitrapyrin (250 mg/kg/day). Following 4 days of exposure to 125 mg/kg/day nitrapyrin in female B6C3F1 mice, the relative liver weight changes (12.6% increase compared to control), panlobular hepatocellular proliferation (1.4-fold increase compared to control), and increased *Cyp2b10* gene expression (11.4-fold compared to control) indicate female mice did not attain a hepatic response of a similar magnitude as male mice exposed to nitrapyrin over the same duration, albeit the dose in male mice was two times higher.

In order to further contextualize the data for nitrapyrin in female mice, particularly given the response in female mice was not as robust as observed for males, data for nitrapryin was compared with rodent MIBK, phenobarbital, and sulfaxoflor. These known CAR activators result in significantly induced *Cyp2b10* transcript levels. Hepatic *Cyp2b10* levels in male mice exposed to nitrapyrin ranged from 350 to 390-fold for exposure durations of 4–14 days with maximal *Cyp2b10* induction of 716 and 1092-fold at 7 and 14-days at 400 mg/kg/day, a dose higher than the tumorigenic dose of 250 mg/kg/day (LaRocca, et al., 2017). The magnitude of *Cyp2b10* and relative liver weight increases observed in females treated with 125 mg/kg/day nitrapyrin was in between responses for males at the non-tumorigenic dose level (75 mg/kg/day) and males at a tumorigenic dose level (≥250 mg/kg/day). For nitrapyrin and the additional three CAR activators investigated in this study, tumorigenic dose levels generally exhibited greater magnitudes of relative liver weight (associative event for CAR MoA) and *Cyp2b10* gene expression changes (key event for CAR MoA) compared to non-tumorigenic dose levels (Figure 3). One contributing factor that may influence the observed range is duration of exposure, which for the four molecules reviewed in this manuscript was from 2 to 90 days. As summarized in Elcombe et al., 2014, shorter durations of exposure (ideally 7 days or less) are appropriate for CAR MoA studies in order to capture the burst of proliferation, which is why the female nitrapyrin study herein was 4 days in duration (Elcombe et al., 2014). While the response observed in female mice following exposure to nitrapyrin at 125 mg/kg/day was less robust than what was previously observed in male mice at 250 mg/kg/day, it was within range of the degree of change observed in mice following exposure to tumorigenic doses of other CAR activators. For example, a moderate increase (31-fold) in *Cyp2b10* was observed in female mice after a 7-days exposure to a tumorigenic dose of sulfoxaflor (Geter et al., 2010). These data indicate that a wide range of *Cyp2b10* gene expression increases occurs following exposure to a tumorigenic dose of a CAR activator, and the response observed for nitrapyrin in female mice at 125 mg/kg/day is within the lower end of this range.

Repeated-dose dietary studies with nitrapyrin at ≥300 mg/kg/day in mice resulted in individual cell necrosis in the liver as well as elevation of serum ALT and AST activity (Yano et al., 2008; LaRocca et al., 2017). While cytotoxicity is a MoA that can lead to non-receptor mediated increased cell proliferation, it is unlikely to be a relevant MoA for nitrapyrin-induced murine hepatocellular tumors in either sex. As detailed in LaRocca et al., 2017, equivocal increases in individual cell necrosis were detected at 125 or 250 mg/kg/day in livers from male mice but were not detected in livers from female mice following exposure to carcinogenic levels of nitrapyrin. Additionally, no significant elevation of ALT or AST activity was detected at the tumorigenic dose levels in livers from female or male mice. The absence of key events necessary to support cytotoxicity as the MoA (focal necrosis, increase serum enzymes) at tumorigenic dose levels supports the conclusion that cytotoxicity is not a viable MoA for nitrapyrin-mediated liver tumors in mice. The (LaRocca, et al., 2017) study also summarizes exclusion of additional alternative MoAs, which is applicable for both male and female mice.

*In vivo* repeat dose experiments with nitrapryin demonstrated effects in addition to CAR-mediated hepatocarcinogenesis. For example, Harderian gland adenomas and forestomach tumors were observed in mice. However, due to structural and physiological differences between mice and humans, nitrapyrin-induced forestomach lesions that occur secondary to local irritation are not considered relevant to humans. Additionally, due to a lack of a clear dose-response and incidence just outside of historical control range, the observed Harderian gland tumors are not considered to be treatment-related. See supplemental material for additional discussion.

This study was designed to minimize animal use to clarify if there were any sex-specific differences for nitrapyin-induced murine hepatocarcinogenesis. Several previous MoA studies were conducted in male mice, including a CAR-KO study which supported the conclusion that CAR was necessary for nitrapyrin exposure to elicit Key Event #2: hepatocellular proliferation (LaRocca, et al., 2017). Male mice were initially investigated due to their increased sensitivity to necrosis at very high dose levels, although the weight of evidence supports the conclusion that cytotoxicity is not theMoA for nitrapyrin-induced mouse liver tumors. The MoA studies completed in male mice included dose-response assessment for key events #1 and #2 (LaRocca et al., 2017). A limitation of the current study in female mice is the assessment of a single, tumorigenic dose of nitrapyrin. However, given the wealth of data available from male mice, the weight of evidence supports CAR activation as the MoA for hepatocarcinogenesis in both sexes. While additional experiments to support the nitrapyrin hepatocarcinogenesis MoA could be conducted in female mice, the wealth of data available in male mice and the focused data generation in female mice strongly supports CAR activation as the MoA in both sexes. Indeed, the study design described herein embraces the “Reduction” feature of the 3Rs for animal welfare: Replacement, Reduction, and Refinement.

In conclusion, MoA data generated from female mice exposed to the tumorigenic dose of nitrapyrin (125 mg/kg/day) indicate CAR activation, as demonstrated by an increase in *Cyp2b10* gene expression, relative liver weight, liver hypertrophy, and hepatocellular proliferation. The increased relative liver weight and *Cyp2b10* gene expression observed in nitrapyrin-exposed female mice were consistent with responses observed in other CAR activators and nitrapyrin male MoA data. Therefore, the data support the conclusion that nitrapyrin-induced liver tumors in female mice are mediated via a CAR MoA.

## Data Availability

The original contributions presented in the study are included in the article/Supplementary Material, further inquiries can be directed to the corresponding author.

## Footnotes

1
^™^ Trademarks of Corteva Agriscience, DuPont or Pioneer, and their affiliated companies or their respective owners.
