# Supplementary material for: Bridging Sex-Specific Differences in the CAR-Mediated Hepatocarcinogenesis of Nitrapyrin Using Molecular and Apical Endpoints
Source: Front Toxicol. 2021 Oct 29;3:766196. doi: 10.3389/ftox.2021.766196 (PMC8915892; doi:10.3389/ftox.2021.766196)
Supplement: Supplementary file 1 [file Table1.DOCX]

**Supplemental Table 1. Individual BrdU Hepatocyte Cell Proliferation Data in Female B6C3F1 Mice Treated with 0 or 125 mg/kg/day Nitrapyrin for 4 days**

| Treatment Groupd | Periportal | | | Midzonal | | | Centrilobular | | | Periportal Positive Labeled % | Midzonal Positive Labeled % | Centrilobular Positive Labeled % | Total Positive Labeled % |
| --- | --- | --- | --- | --- | --- | --- | --- | --- | --- | --- | --- | --- | --- |
|  | Positive Cells | Negative Cells | Total | Positive Cells | Negative Cells | Total | Positive Cells | Negative Cells | Total |  |  |  |  |
| 0 mg/kg/day | 102 | 1092 | 1194 | 52 | 1002 | 1054 | 62 | 1035 | 1097 | 8.5 | 4.9 | 5.7 | 6.5 |
| 0 mg/kg/day | 142 | 1221 | 1363 | 117 | 955 | 1072 | 83 | 1110 | 1193 | 10.4 | 10.9 | 7.0 | 9.4 |
| 0 mg/kg/day | 93 | 1163 | 1256 | 32 | 1075 | 1107 | 50 | 1012 | 1062 | 7.4 | 2.9 | 4.7 | 5.1 |
| 0 mg/kg/day | 67 | 962 | 1029 | 81 | 967 | 1048 | 83 | 930 | 1013 | 6.5 | 7.7 | 8.2 | 7.5 |
| 0 mg/kg/day | 65 | 1338 | 1403 | 62 | 1225 | 1287 | 84 | 1440 | 1524 | 4.6 | 4.8 | 5.5 | 5.0 |
| 0 mg/kg/day | 75 | 977 | 1052 | 78 | 994 | 1072 | 77 | 1040 | 1117 | 7.1 | 7.3 | 6.9 | 7.1 |
| 125 mg/kg/day | 115 | 922 | 1037 | 74 | 930 | 1004 | 86 | 939 | 1025 | 11.1 | 7.4 | 8.4 | 9.0 |
| 125 mg/kg/day | 105 | 1369 | 1474 | 78 | 1058 | 1136 | 71 | 1017 | 1088 | 7.1 | 6.9 | 6.5 | 6.9 |
| 125 mg/kg/day | 130 | 1103 | 1233 | 86 | 980 | 1066 | 94 | 1132 | 1226 | 10.5 | 8.1 | 7.7 | 8.8 |
| 125 mg/kg/day | 182 | 1265 | 1447 | 117 | 1005 | 1122 | 94 | 988 | 1082 | 12.6 | 10.4 | 8.7 | 10.8 |
| 125 mg/kg/day | 190 | 1358 | 1548 | 146 | 1060 | 1206 | 134 | 1084 | 1218 | 12.3 | 12.1 | 11.0 | 11.8 |
| 125 mg/kg/day | 211 | 1806 | 2017 | 174 | 1645 | 1819 | 142 | 1516 | 1658 | 10.5 | 9.6 | 8.6 | 9.6 |

Each row represents an individual animal.

**Supplemental Table 2. Comparison of Relative Liver Weight Change and *Cyp2b10* gene expression from MIBK, Sulfoxaflor, Phenobarbital, and Nitrapyrin Studies.**

| **Molecule** | **Strain** | **Sex** | **Dose Level**  **(ppm or mg/kg/day)** | **Exposure Time (Days)** | **Relative Liver Weight Change (%)** | ***Cyp2b10* Fold Change** |
| --- | --- | --- | --- | --- | --- | --- |
| MIBK | B6C3F1 | Male | 1800 ppm | 10 | 14 | 982 |
| MIBK | B6C3F1 | Female | 1800 ppm | 10 | 23 | 234 |
| MIBK | C57Bl/6 | Male | 1800 ppm | 10 | 8 | 599 |
| MIBK | C57Bl/6 | Female | 1800 ppm | 10 | 19 | 270 |
| Sulfoxaflor | CD1 | Male | 500 ppm | 7 | 4 | 42 |
| Sulfoxaflor | CD1 | Male | 750 ppm | 7 | 17 | 55 |
| Sulfoxaflor | CD1 | Female | 1000 ppm | 7 | 38 | 20 |
| Sulfoxaflor | CD1 | Female | 1500 ppm | 7 | 43 | 31 |
| Sulfoxaflor | CD1 | Male | 300 ppm | 28 | 11 | 62 |
| Sulfoxaflor | CD1 | Female | 1500 ppm | 28 | 19 | 94 |
| Sulfoxaflor | CD1 | Male | 750 ppm | 90 | 25 | 57 |
| Sulfoxaflor | CD1 | Female | 1500 ppm | 90 | 40 | 54 |
| Sulfoxaflor | C57Bl/6 | Male | 750 ppm | 7 | 25 | 119 |
| Phenobarbital | CD1 | Male | 0.15 mg/kg/day | 2 | 0 | 1 |
| Phenobarbital | CD1 | Male | 1.5 mg/kg/day | 2 | 0 | 1 |
| Phenobarbital | CD1 | Male | 15 mg/kg/day | 2 | 0 | 65 |
| Phenobarbital | CD1 | Male | 75 mg/kg/day | 2 | 3 | 168 |
| Phenobarbital | CD1 | Male | 150 mg/kg/day | 2 | 20 | 522 |
| Phenobarbital | CD1 | Male | 0.15 mg/kg/day | 7 | 0 | 1 |
| Phenobarbital | CD1 | Male | 1.5 mg/kg/day | 7 | 6 | 3 |
| Phenobarbital | CD1 | Male | 15 mg/kg/day | 7 | 2 | 36 |
| Phenobarbital | CD1 | Male | 75 mg/kg/day | 7 | 22 | 166 |
| Phenobarbital | CD1 | Male | 150 mg/kg/day | 7 | 48 | 474 |
| Phenobarbital | CD1 | Female | 0.15 mg/kg/day | 2 | 0 | 1 |
| Phenobarbital | CD1 | Female | 1.5 mg/kg/day | 2 | 0 | 2 |
| Phenobarbital | CD1 | Female | 15 mg/kg/day | 2 | 0 | 14 |
| Phenobarbital | CD1 | Female | 75 mg/kg/day | 2 | 4 | 167 |
| Phenobarbital | CD1 | Female | 150 mg/kg/day | 2 | 21 | 155 |
| Phenobarbital | CD1 | Female | 0.15 mg/kg/day | 7 | 8 | 1 |
| Phenobarbital | CD1 | Female | 1.5 mg/kg/day | 7 | 0 | 1 |
| Phenobarbital | CD1 | Female | 15 mg/kg/day | 7 | 8 | 12 |
| Phenobarbital | CD1 | Female | 75 mg/kg/day | 7 | 26 | 74 |
| Phenobarbital | CD1 | Female | 150 mg/kg/day | 7 | 32 | 169 |
| Nitrapyrin | B6C3F1 | Male | 75 mg/kg/day | 7 | 3 | 4 |
| Nitrapyrin | B6C3F1 | Male | 250 mg/kg/day | 7 | 19 | 351 |
| Nitrapyrin | B6C3F1 | Male | 400 mg/kg/day | 7 | 39 | 716 |
| Nitrapyrin | B6C3F1 | Male | 75 mg/kg/day | 14 | 3 | 4 |
| Nitrapyrin | B6C3F1 | Male | 250 mg/kg/day | 14 | 24 | 390 |
| Nitrapyrin | B6C3F1 | Male | 400 mg/kg/day | 14 | 49 | 1092 |
| Nitrapyrin | B6C3F1 | Male | 250 mg/kg/day | 4 | 27 | 370 |
| Nitrapyrin | B6C3F1 | Male | 250 mg/kg/day | 7 | 34 | 563 |
| Nitrapyrin | C57bl/6NTac | Male | 250 mg/kg/day | 4 | 28 | 240 |
| Nitrapyrin | C57bl/6NTac | Male | 250 mg/kg/day | 7 | 30 | 169 |
| Nitrapyrin | B6C3F1 | Female | 150 mg/kg/day | 4 | 13 | 11 |
